# Human placental uptake of glutamine and glutamate is reduced in fetal growth restriction

**DOI:** 10.1038/s41598-020-72930-7

**Published:** 2020-10-01

**Authors:** Kirsty R. McIntyre, Kirsty M. M. Vincent, Christina E. Hayward, Xiaojia Li, Colin P. Sibley, Michelle Desforges, Susan L. Greenwood, Mark R. Dilworth

**Affiliations:** 1 Maternal and Fetal Health Research Centre, Division of Developmental Biology and Medicine, School of Medical Sciences, Faculty of Biology, Medicine and Health, The University of Manchester, Manchester, UK; 2 Manchester Academic Health Science Centre, St. Mary’s Hospital, Manchester University NHS Foundation Trust, Manchester, UK; 3 School of Medicine, Dentistry and Nursing, College of Medical, Veterinary and Life Sciences, University of Glasgow, Wolfson Medical School Building, University Avenue, Glasgow, G12 8QQ UK

**Keywords:** Physiology, Reproductive biology, Immunoblotting, PCR-based techniques

## Abstract

Fetal growth restriction (FGR) is a significant risk factor for stillbirth, neonatal complications and adulthood morbidity. Compared with those of appropriate weight for gestational age (AGA), FGR babies have smaller placentas with reduced activity of amino acid transporter systems A and L, thought to contribute to poor fetal growth. The amino acids glutamine and glutamate are essential for normal placental function and fetal development; whether transport of these is altered in FGR is unknown. We hypothesised that FGR is associated with reduced placental glutamine and glutamate transporter activity and expression, and propose the mammalian target of rapamycin (mTOR) signaling pathway as a candidate mechanism. FGR infants [individualised birth weight ratio (IBR) < 5th centile] had lighter placentas, reduced initial rate uptake of ^14^C-glutamine and ^14^C-glutamate (per mg placental protein) but higher expression of key transporter proteins (glutamine: LAT1, LAT2, SNAT5, glutamate: EAAT1) versus AGA [IBR 20th–80th]. In further experiments, in vitro exposure to rapamycin inhibited placental glutamine and glutamate uptake (24 h, uncomplicated pregnancies) indicating a role of mTOR in regulating placental transport of these amino acids. These data support our hypothesis and suggest that abnormal glutamine and glutamate transporter activity is part of the spectrum of placental dysfunction in FGR.

## Introduction

Fetal growth restriction (FGR), which affects approximately 5% of pregnancies, refers to the inability of a fetus to achieve its growth potential and is primarily caused by placental dysfunction^1^. FGR is a major risk factor for stillbirth and neonatal, childhood and adulthood morbidity^2–5^. Despite the significant societal and economic burden of FGR^6–8^ there are currently no approved pharmacological treatments available^9^. The reticence to develop novel therapies for placental dysfunction in part reflects concerns of potential fetal toxicity but is also influenced by an incomplete understanding of placental function in health and disease^10^. FGR is often associated with one, or a combination of, the following characteristics: small placental size, abnormal placental structure, abnormal uteroplacental and fetoplacental blood flow, and abnormal function of the syncytiotrophoblast, the transporting epithelium of the placenta^1,11–15^. Inconsistencies in the definition of FGR prompted a recent Delphi study, which recommended a consensus-based clinical definition of FGR^16^.

In women, FGR is associated with reduced placental activity (per mg membrane protein) of a number of amino acid transporter systems including systems A and L^13–15,17^. Furthermore, in a rat model of FGR, reduced placental amino acid transport by system A is evident prior to the reduction in fetal growth^18^, whilst inhibition of placental system A directly results in reduced fetal growth^19^, demonstrating that a reduction of placental amino acid provision by system A can cause FGR.

The amino acids glutamine and glutamate are essential for pH homeostasis, nucleotide synthesis and protein anabolism^20,21^. Glutamine is a non-essential amino acid that becomes conditionally essential during pregnancy as fetal demand exceeds maternal synthesis^22,23^. Demand is met through interorgan recycling of glutamine and glutamate. Deamination of glutamine in the fetal liver produces glutamate, an important nitrogen resource and precursor of γ-amino butyric acid (GABA), a key inhibitory neurotransmitter^23–25^. Glutamate is transported across the syncytiotrophoblast microvillous membrane (MVM: maternal facing) and basal membrane (BM: fetal facing) by high affinity Excitatory Amino Acid Transporters (EAATs; system X_AG-_)^26^ and is converted to glutamine in the placenta^27^. Glutamine is a substrate of the amino acid transporter systems A (isoforms SNAT1, 2 and 4), N (SNAT5), L (LAT1 and 2), y^+^L and ASC^21^. However, system y^+^L-mediated glutamine transfer across the MVM is negligible and system ASC isoforms are predominantly localised to the BM^28^.

There are as yet no studies of placental glutamine and glutamate transport in the placenta in human FGR but, as noted above, substantial evidence to show that system A and system L transporter activity is reduced in this condition^13–15,17^. The reasons behind the reduction in placental system A and system L activity in FGR are poorly understood, not least because the determinants of appropriate provision of amino acids to the fetus in normal pregnancy have not been adequately defined. However, there is evidence that the mammalian target of rapamycin (mTOR) signaling pathway, specifically mTOR complex 1 (mTORC1), regulates system A and L activity through reduced plasma membrane trafficking of SNAT2 in the case of system A^29,30^ and that placental mTOR activity, as evidenced by reduction in the important downstream signaling molecule phosphorylated ribosomal S6 kinase (phospho-S6K1), is reduced in FGR^29,31^. The mTOR pathway, proposed to act as a nutrient sensor, is thus a candidate mechanism by which glutamine and glutamate transport is modulated in normal pregnancy, though this has never been tested.

In the current investigation we tested the hypotheses that (a) placental glutamine and glutamate transporter expression and activity is reduced in FGR infants compared with infants appropriately grown for gestational age (AGA), and (b) inhibiting mTOR activity in placentas from uncomplicated pregnancies would result in a reduction in placental uptake of glutamine and glutamate.

## Results

### Transporter-mediated uptake of ^14^C-glutamine, ^14^C-glutamate and ^14^C-MeAIB is reduced in FGR pregnancies

**Figure 1:**
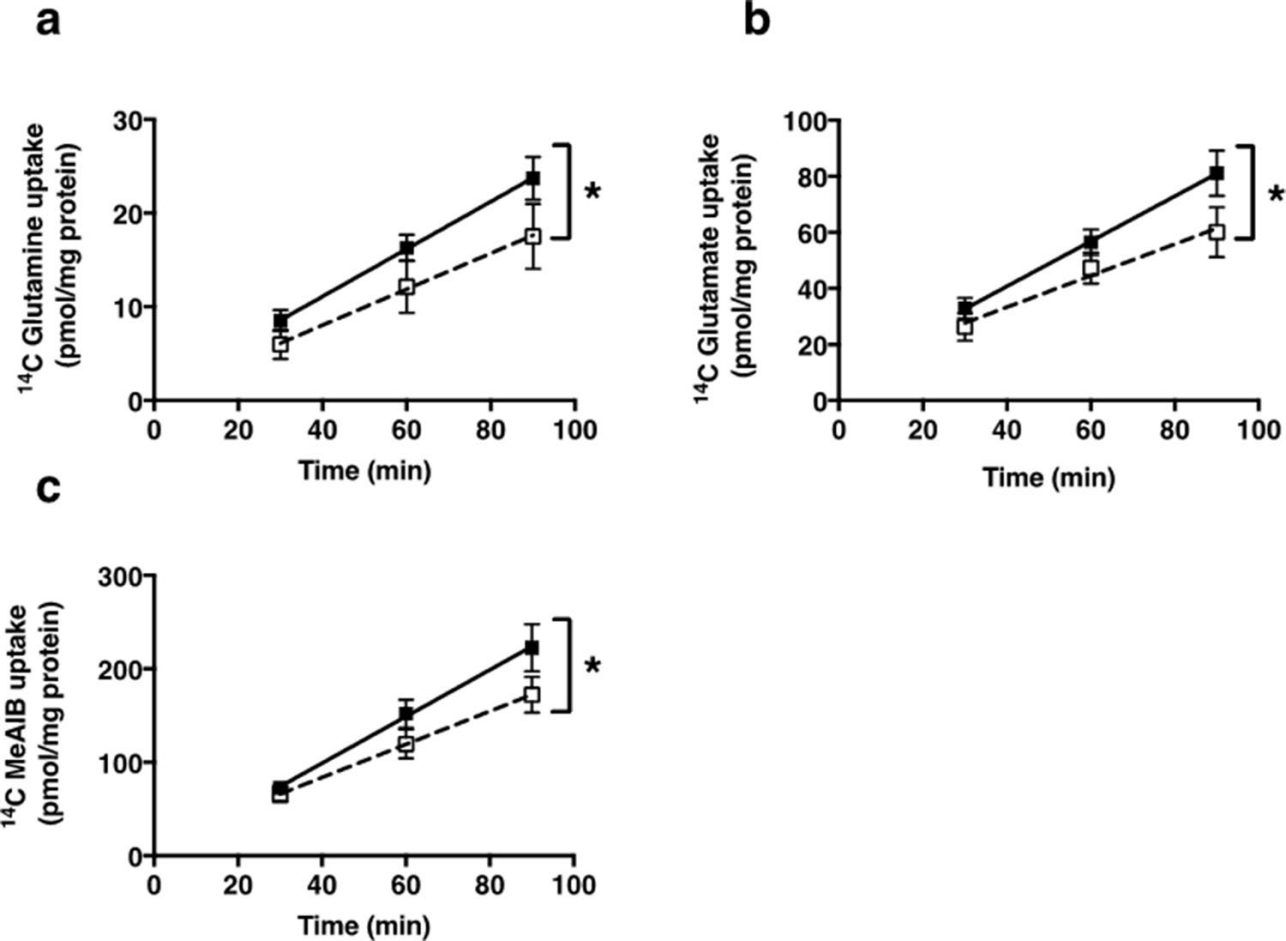
Transporter-mediated uptake of ^14^C-glutamine (**a**), ^14^C-glutamate (**b**) and ^14^C-MeAIB (**c**) by placental villous fragments from AGA infants (n = 13–14, solid symbols) and FGR (n = 10–11, hollow symbols). Data are mean ± SEM * *P* < 0.05 Linear regression analysis showed a significant difference in elevation for glutamine (*P* < 0.02), glutamate (*P* < 0.02) and MeAIB (*P* < 0.03) uptake in FGR versus AGA.

A summary of the demographics of study participants is shown in Table 1. FGR infants weighed significantly less than AGA infants (*P* < 0.001) and had significantly lighter placentas (*P* < 0.001). There was a difference in the gestational age of FGR babies compared with AGA babies (Table 1; *P* < 0.05). Transporter-mediated uptake of ^14^C-glutamine, ^14^C-glutamate (validation of method: Supplementary Fig. 1) and ^14^C-MeAIB at initial rate was significantly lower in placentas of FGR babies (IBR < 5th centile) compared with placentas of AGA babies (IBR 20th–80th centile, *P* < 0.05) (Fig. 1). There was no effect of gestational age on amino acid uptake (Supplementary Fig. 2).

**Table 1:** Maternal and fetal demographics from AGA and FGR cohorts.

	AGA (n = 14)	FGR (n = 11)	*P* value
Maternal age (years)	34 (23–40)	32 (25–39)	0.946
Body mass index (kg/m^2^)	24.9 (18.8–31.2)	23.9 (19.8–33.9)	0.442
Birth weight (g)	3355 (2820–3960)	1730 (717–2860)	< 0.001
Trimmed placental weight (g)	498.1 (402.3–719.4)	301.6 (159.5–429.3)	< 0.001
Individualised birth weight ratio (IBR)	53.6 (26.4–79.2)	1 (0–3.5)	< 0.001
Gestation (weeks + days)	39 + 0 (37 + 1–40 + 2)	35.2 (29 + 1–40 + 4)	0.049
Mode of delivery	ELCS (89%)	ELCS (55%)	0.623
	NVD (11%)	EMCS (18%)	
		NVD (27%)	
Parity	1 (0–8)	1 (0–5)	0.315
Gravidity	2 (1–12)	2 (1–6)	0.971
Ethnicity	Caucasian (71%)	Caucasian (64%)	0.999
Smoking status (yes/no)	No (100%)	No (73%)	0.072

Data are median (range) or percentage of total. Analysis by Mann–Whitney test or by Fishers exact test (mode of delivery, ethnicity and smoking status). *ELCS* elective caesarean section, *EMCS* emergency caesarean section, *NVD* normal vaginal delivery.

**Figure 2:**
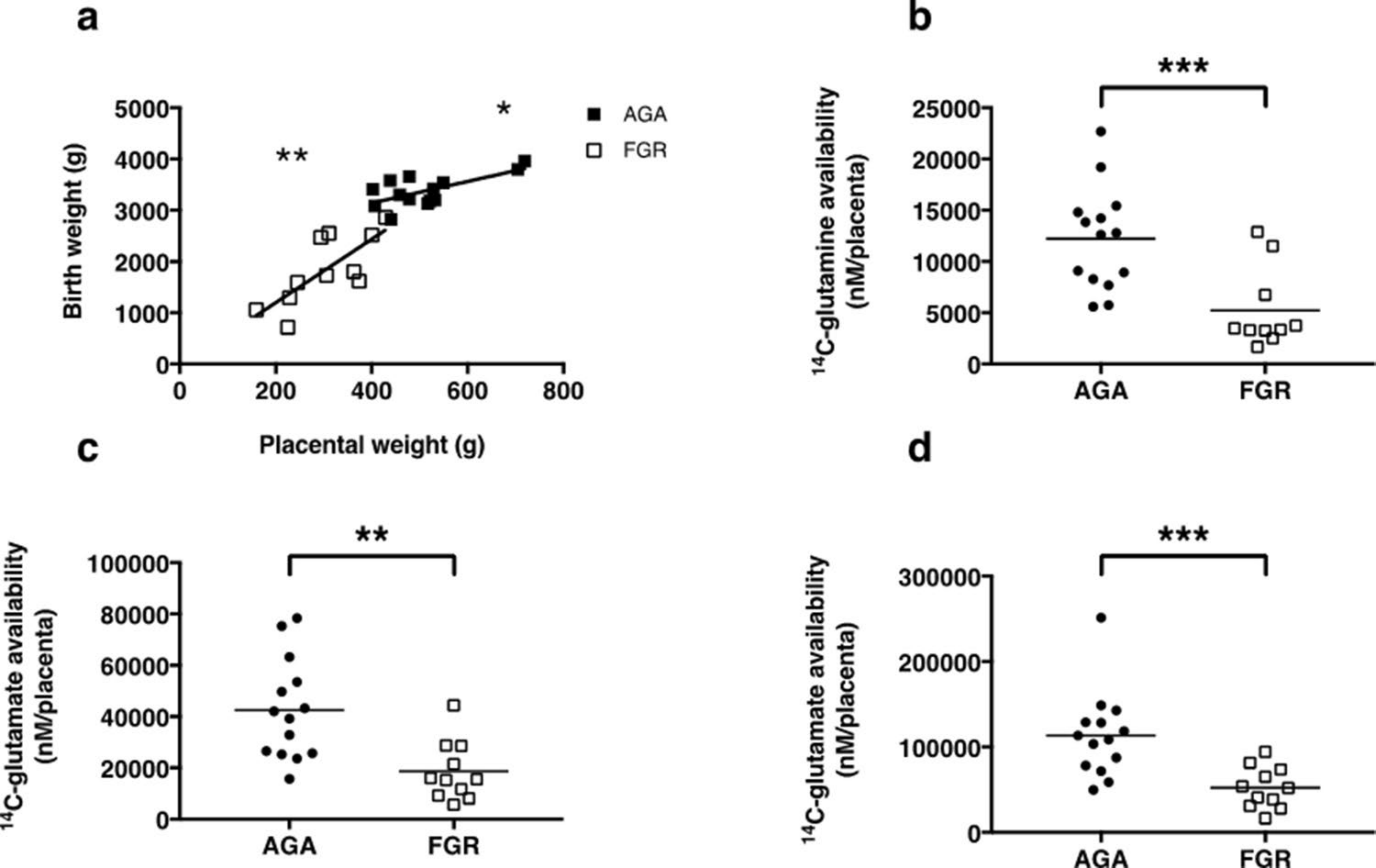
Correlation between trimmed placental weight and birth weight (**a**) for AGA (n = 14, solid symbols) and FGR infants (n = 11, hollow symbols) ** *P* < 0.01, * *P* < 0.05: Linear regression. Availability of ^14^C-glutamine (**b**), ^14^C-glutamate (**c**) and ^14^C-MeAIB (**d**) for delivery to the fetus in placentas from AGA compared with FGR infants. *** *P* < 0.001, ** *P* < 0.01: Mann–Whitney test.

Linear regression analyses fitted to all data indicated that transporter-mediated uptake of ^14^C-glutamine at 90 min was correlated with uptake of ^14^C-MeAIB (*P* < 0.05) and ^14^C-glutamate (*P* < 0.001, Supplementary Fig. 3). However, when analyses were fitted to either AGA or FGR groups, we found that the correlation between ^14^C-glutamine and ^14^C-MeAIB only remained for placentas from FGR babies (*P* < 0.05), highlighting the importance of system A in these pregnancies. ^14^C-glutamine and ^14^C-glutamate uptake was correlated for both AGA and FGR infants, which suggests that there is an intrinsic relationship between the uptake of these two amino acids that is not altered in FGR.

**Figure 3:**
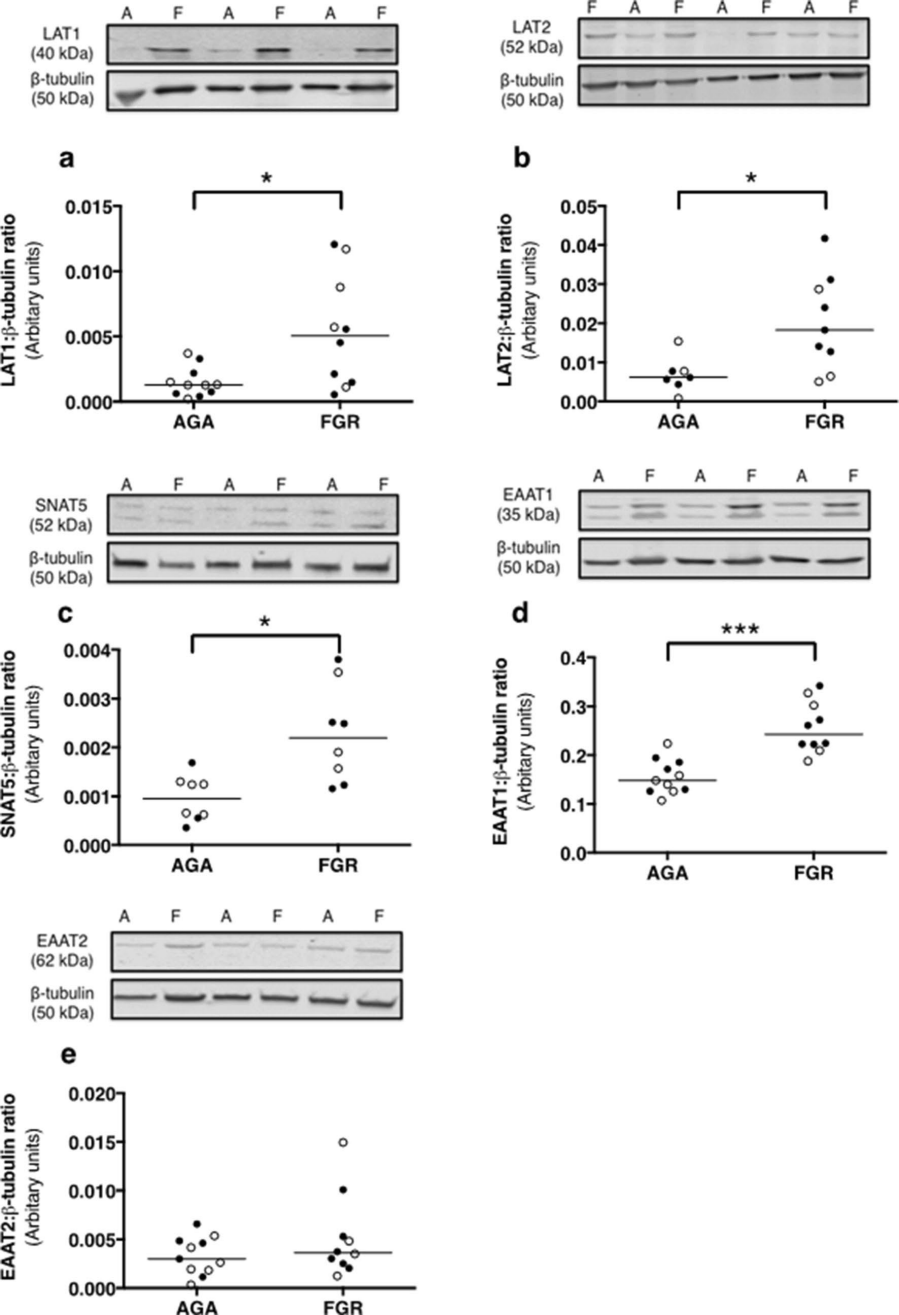
Expression of glutamine [LAT1 (**a**), LAT2 (**b**), SNAT5 (**c**)] and glutamate [EAAT1 (**d**), EAAT2 (**e**)] transporter proteins in membrane-enriched placental homogenates from AGA (n = 7–11) and FGR (n = 8–10) infants. Densitometric analysis is expressed as a ratio of β-tubulin signal. Line denotes median, *** *P* < 0.001, * *P* < 0.05: Wilcoxon signed-rank test.

### Total amino acid availability for transfer from the placenta to the fetus is reduced in FGR versus AGA infants

Trimmed placental weight and birth weight were correlated for both AGA (*P* < 0.05) and FGR (*P* < 0.01) infants (Fig. 2A). A proxy measure of the availability of amino acids (^14^C-glutamine, ^14^C-glutamate and ^14^C-MeAIB) for transfer from the syncytiotrophoblast to the fetus [trimmed placental weight (g) × amino acid uptake at 90 min (per mg placental villous fragment protein)] was significantly lower for placentas of FGR versus AGA infants (Fig. 2B–D).

### Placental transporter protein and mRNA expression is altered in FGR versus AGA infants

Expression of glutamine transporter proteins (system L: LAT1, 2, and system N: SNAT5) was significantly higher in placentas from FGR (IBR 0.0–3.5) versus AGA (IBR 27.9–79.1) pregnancies (Fig. 3A–C, P < 0.05). Expression of EAAT1 (glutamate is a substrate) was significantly higher in placentas from FGR versus AGA infants (Fig. 3D, P < 0.001). EAAT2 expression was not different between groups (Fig. 3E). Expression of β-tubulin was not different between AGA and FGR groups. Full-length blots are presented in Supplementary Fig. 4.

**Figure 4:**
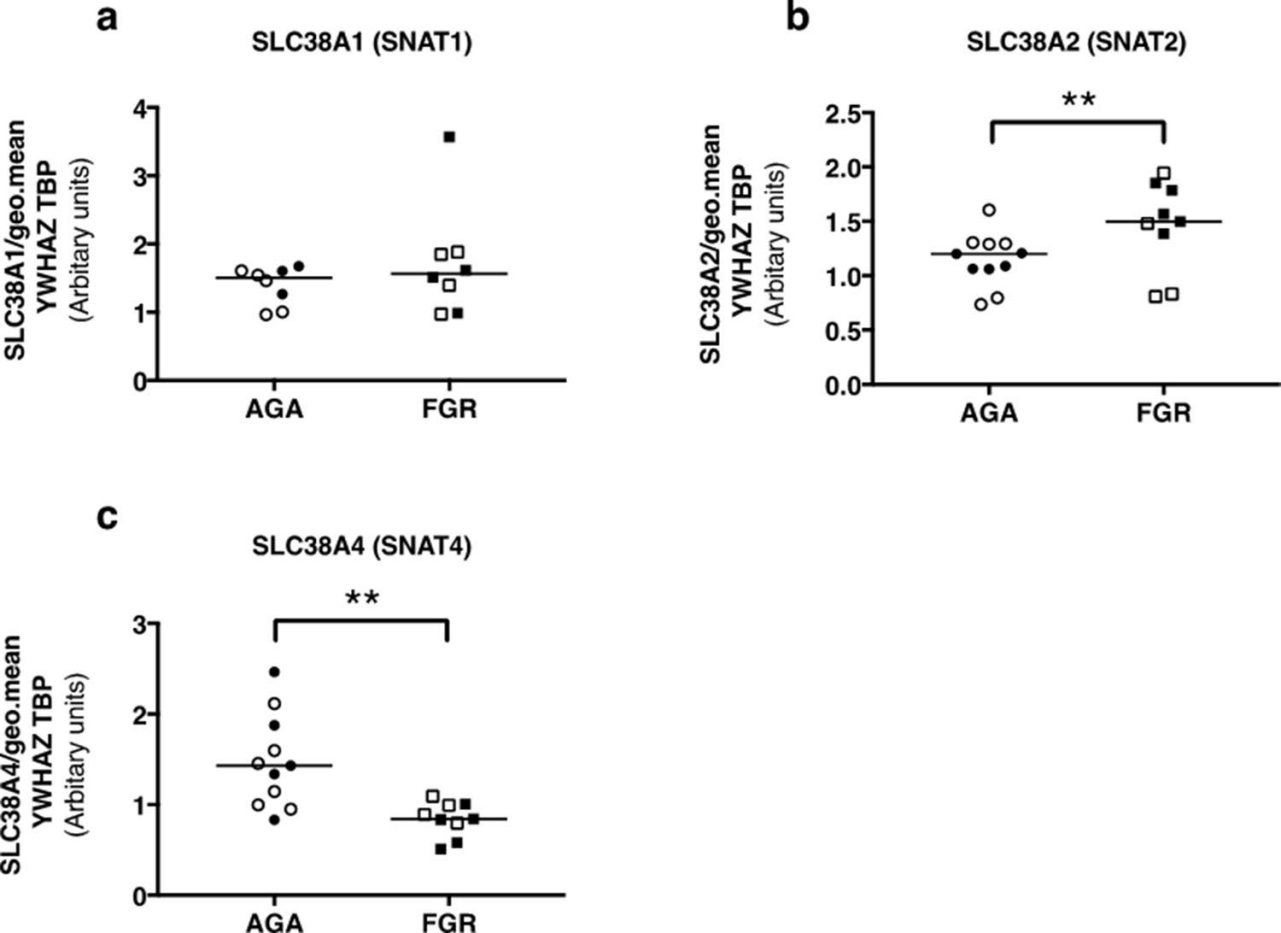
mRNA expression of *SLC38A1* (**a**), *SLC38A2* (**b**) and *SLC38A4* (**c**) in placentas from AGA (n = 8–11) and FGR (n = 8–9) placentas, normalised to the geometric mean of *YWHAZ* and *TBP*. ** *P* < 0.01: Mann–Whitney test.

It was not possible to assess protein expression of system A transporter isoforms (SNAT1, 2 and 4), as there were no suitably validated commercially available antibodies. However, expression of the gene that encodes the system A isoform SNAT2 (*SLC38A2*) was significantly higher in placentas from FGR than AGA babies (*P* < 0.01) (Fig. 4B). Conversely, *SLC38A4* mRNA expression (encodes SNAT4) was significantly lower in placentas from FGR versus AGA pregnancies (*P* < 0.01) (Fig. 4C); expression of *SLC38A1* (encodes SNAT1) was not significantly different between placentas of AGA and FGR infants (Fig. 4A).

### Rapamycin inhibits steady state accumulation of ^14^C-MeAIB, ^14^C-glutamine and ^14^C-glutamate by placental fragments from uncomplicated pregnancies

**Figure 5:**
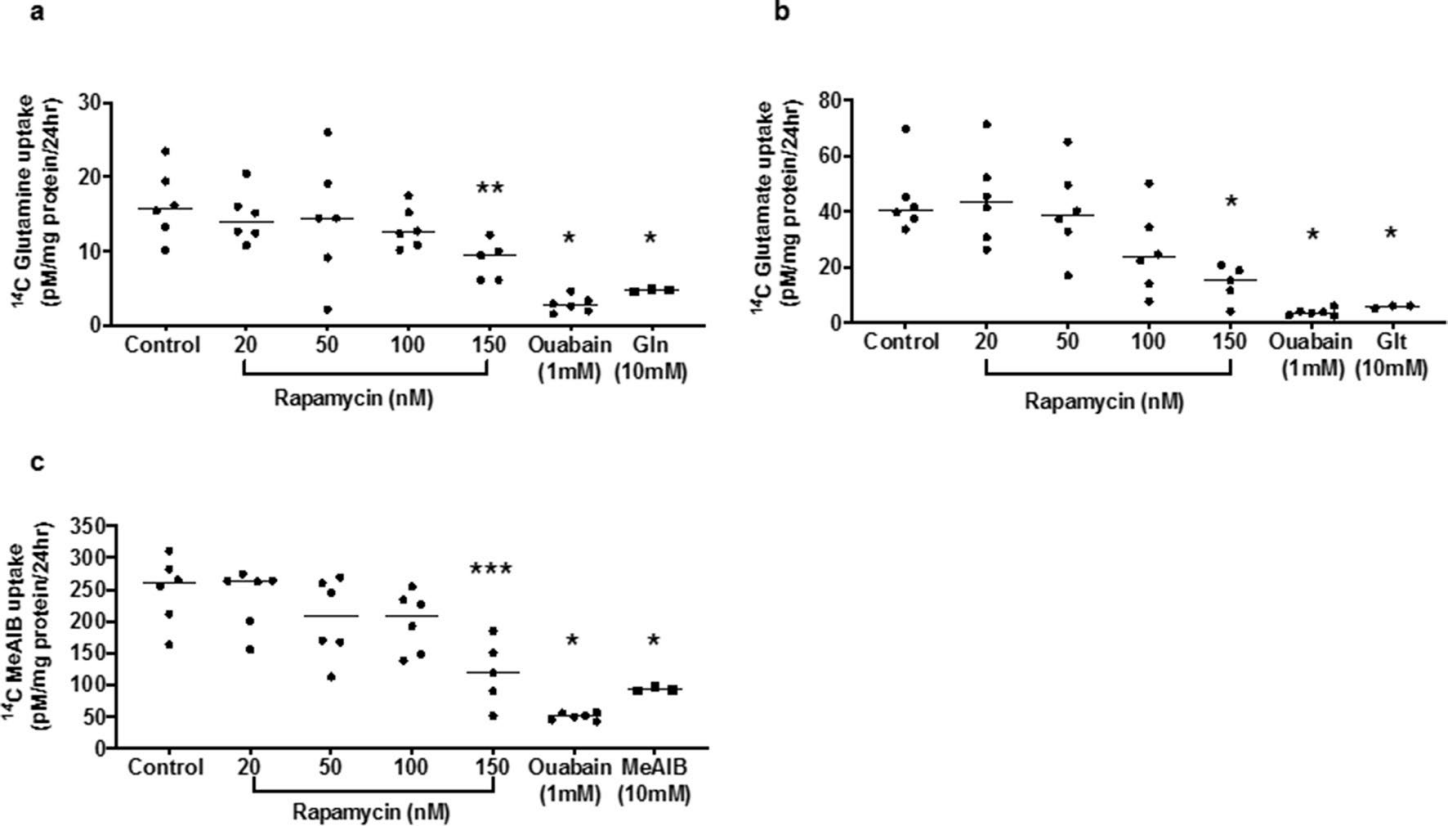
Effect of rapamycin on ^14^C-MeAIB (**a**) ^14^C-glutamine (**b**) and ^14^C-glutamate (**c**) uptake into placental villous explants (n = 6) over 24 h. *** *P* < 0.001, ** *P* < 0.01, * *P* < 0.05 versus control: ANOVA with Dunn’s multi-comparison post hoc test. 1 mM ouabain (inhibitor of Na^+^/K^+^-ATPase) reduced uptake of all radiolabelled amino acids by 70–85% (* *P* < 0.03: Wilcoxon matched pairs; n = 6 placentas). 10 mM unlabelled amino acids [MeAIB (**a**) Gln = glutamine (**b**) Glt = glutamate (**c**); competing substrate] significantly reduced uptake of the corresponding radiolabelled amino acid (* *P* < 0.02: Mann–Whitney; n = 3 placentas). Line denotes median.

Rapamycin caused a concentration-dependent inhibition of ^14^C-MeAIB, ^14^C-glutamine and ^14^C-glutamate uptake by placental villous explants from uncomplicated pregnancies (IBR 20–70) over 24 h, which was significantly lower than control at 150 nM (Fig. 5; ^14^C-MeAIB *P* < 0.001; ^14^C-glutamate *P* < 0.01; ^14^C-glutamine *P* < 0.05 versus control). Uptake of the radiolabelled amino acids was reduced to 15–30% (range) of control by the Na^+^/K^+^-ATPase inhibitor ouabain, indicating that at least 70% of radiolabel accumulation by the tissue over 24 h could be attributed to amino acid transporter activity, driven by an inwardly directed Na^+^ gradient. Neither rapamycin nor ouabain altered the uptake of ^14^C-3-0-methylglucose (3-0MG) over 24 h (Supplementary Fig. 5). The transporter-mediated uptake of ^14^C-MeAIB, ^14^C-glutamine and ^14^C-glutamate was also reduced by a high concentration (10 mM) of unlabelled MeAIB, glutamine and glutamate to 35%, 28% and 13% of control respectively (Fig. 5).

### Phospho-S6K1 but not total S6K1 expression is inhibited by rapamycin

**Figure 6:**
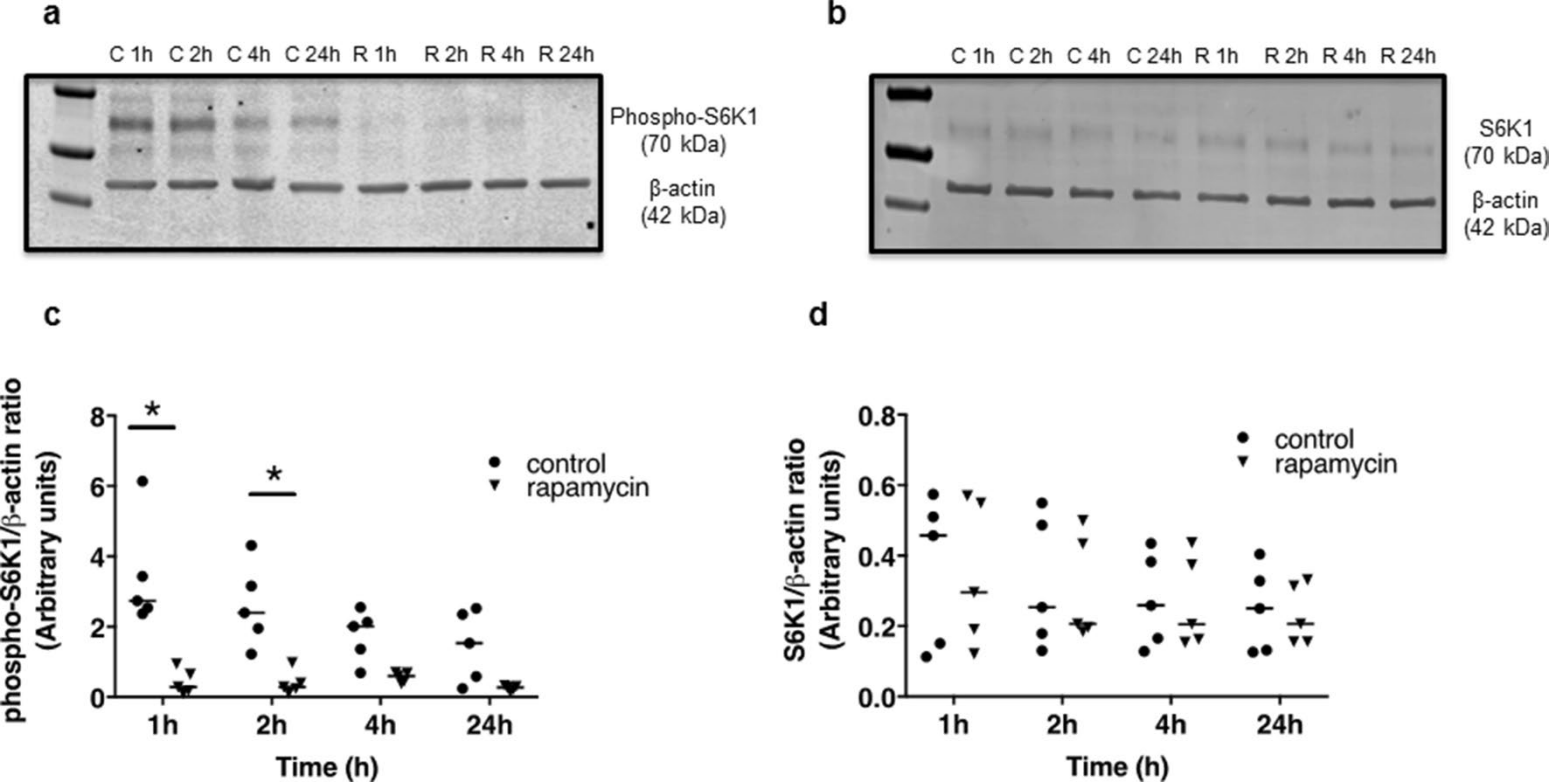
Representative western blots of phospho-S6K1 (**a**) and total S6K1 (**b**) protein in placental villous explants treated for 1, 2, 4 and 24 h with (**R**) or without (control, **C**) 150 nM rapamycin. Densitometric analysis is expressed as a ratio of β-actin signal. Data are shown in (**C**) and (**D**) (n = 5 placentas). Line denotes median, * *P* < 0.05: 2-way repeated measures ANOVA with Sidak test.

Both time and exposure to rapamycin had an overall effect in reducing placental (uncomplicated pregnancies; IBR 37.1–96.6) expression of phosphorylated ribosomal S6 kinase (phospho-S6K1, Fig. 6A,C). When comparing groups directly at each timepoint, expression of phospho-S6K1 was lower in the rapamycin versus control groups at 1 and 2 h (*P* < 0.05) with a trend towards significance at 4 h (*P* = 0.07, Fig. 6C). Rapamycin had no effect on total S6K1 expression at any timepoint (Fig. 6B,D). Full-length blots are presented in Supplementary Fig. 6.

## Discussion

In agreement with our hypothesis this study shows that initial rate uptake of glutamine and glutamate, important amino acids for fetal growth and placental metabolism, into placental villous fragments is reduced in FGR. This reduction was not accompanied by reduced expression of transporters utilised by glutamine and glutamate. To the contrary, expression of the transporter proteins, LAT1, LAT2, SNAT5 and EAAT1 were increased in placental homogenates of FGR versus AGA infants. Reasons for this disparity are not known at present but possibilities are discussed below.

In both AGA and FGR, we estimate that approximately twice as much glutamate as glutamine is transported across the MVM (taking into account that different concentrations of these radiolabeled amino acids were used for the initial rate uptake experiments: see Methods). This is probably reflective of the fact that glutamate is readily metabolised to glutamine in the cytosol of the syncytiotrophoblast for subsequent transfer to the fetus and reinforces the importance of glutamate, albeit indirectly, for fetal growth as previously suggested in mice^32^. Worthy of note is that measures of ^14^C uptake in these studies are unable to discriminate between glutamine and glutamate should interconversion have taken place. If interconversion did occur over the time course of the experiment (30–90 min), the studies show that there is a reduction in ^14^C labelled substrates, important for both placental metabolism and fetal growth, in FGR versus AGA.

In the current study we have also shown that, in uncomplicated pregnancies, exposure to rapamycin inhibits placental glutamine and glutamate uptake at steady state. The involvement of mTOR in this effect is evidenced by the reduction in phospho-, but not total, S6K1 expression following rapamycin exposure. Experiments were performed to assess the contribution of transporter-mediated glutamine and glutamate uptake to the total accumulation over 24 h. Using a high concentration of unlabelled substrates to compete for binding sites on the transporters, or ouabain to reduce the activity of transporters primarily- and secondarily-dependent on the intracellular Na^+^ gradient (latter include exchangers such as system L, which rely on intracellular substrates that are taken up by Na^+^ dependent transporters), we showed that a maximum of 30% of amino acid uptake over 24 h could be by non-specific diffusion (e.g. through the lipid bilayer or damaged areas of tissue). Accordingly, it is likely that rapamycin reduced MeAIB, glutamine and glutamate uptake by inhibiting the activity of the transporter systems A, L and X_AG-_. This effect is consistent with our hypothesis and with the regulation of system A and L transporter activity by mTOR reported previously^30^.

Our data are consistent with existing reports in the literature that FGR fetuses have smaller placentas than AGA babies^1^ and demonstrate reduced transporter-mediated uptake of ^14^C-MeAIB (system A activity)^13–15,33^. Placental uptake of amino acids is important for their subsequent delivery to the fetus and also for modifying the intracellular pool of amino acids available for exchange, for example by system L transporters^34^. One may expect a smaller placenta (by weight) to have less surface area available for exchange. Indeed in FGR, the surface area of terminal villi and capillaries is decreased^35^. Ideally, surface area available for nutrient transport would be used as the denominator by which to calculate amino acid transporter activity. However, in the absence of a method to accurately measure syncytiotrophoblast MVM surface area for this assay, placental uptake was measured per mg protein as a proxy measure of placental size. Reduced amino acid uptake (per mg placental protein) in combination with a smaller placenta theoretically contributes to reduced amino acid availability for delivery to the fetus, as illustrated by the proxy measure of total amino acid within the syncytiotrophoblast available for exchange (Fig. 2B–D). It is also postulated that a reduction in initial rate uptake of glutamine and glutamate will lead to a lower intracellular concentration in placentas of FGR compared with AGA pregnancies^36^. Reductions in amino acid availability thus offer a rationale as to why fetal growth may be compromised in FGR. Further work is required to establish the impact of reduced amino acid uptake in FGR on the composition of amino acid pools within the placenta.

A limitation of the study was the significant difference in the gestation of FGR samples compared with AGA (Table 1). However, analyses performed confirmed no difference in uptake of radiolabeled amino acids from preterm compared to term delivery (Supplementary Fig. 2).

Transporter abundance was assessed as a potential mechanistic explanation for the reduction in amino acid transporter activity in FGR. However, the data presented here show a disparity between transporter activity and expression. In FGR, placental expression of LAT1, LAT2, SNAT5 and EAAT1 transporter proteins was higher compared with AGA. This is in agreement with previous reports that LAT1 expression, as analysed by immunohistochemistry, is increased in the MVM of the syncytiotrophoblast in FGR^37^. This is the first study to show that placental system N and X_AG-_ (SNAT5 and EAAT1) protein expression is also elevated in FGR.

Assessment of system A transporter proteins by Western blotting was not conducted in the current study due to an absence of commercially available and validated antibodies that reliably produce a specific signal. Instead, we evaluated mRNA expression and found that placental *SLC38A2* (SNAT2) expression was higher and *SLC38A4* (SNAT4) expression lower in FGR compared with AGA pregnancy. These data conflict with previous observations from a similar study cohort in humans^31^ (FGR < 3rd centile versus AGA 25th–75th centile, non-customised growth charts) that reported reduced SNAT1 and 2, and no difference in SNAT4, protein expression in MVM isolates from FGR compared with AGA infants; no differences were found when whole homogenates was compared. Furthermore, a study by Malina et al*.*, compared* SLC38A1* and *SLC38A2* mRNA expression between placentas of women with SGA (< 10th centile) and normal birth weight infants and demonstrated no difference between groups^38^. Experiments in animal models yield conflicting results. Placental SNAT1 and 2 expression is reduced in the MVM of a protein restricted rat model of FGR but not in a protein-restricted baboon model^39,40^. In the placental-specific Igf2 knockout mouse, a well characterized model of FGR, placental expression of *Slc38a4* is elevated compared with wild-type mice earlier in pregnancy (embryonic day 16) but normalizes near term whilst expression of *Slc38a1* and *Slc38a2* are no different at either gestational time point^41^. The mechanisms that underpin these disparate observations remain unclear but likely relate to species differences and also the different perturbations to induce FGR in the animal models. In the current study,* SLC38A1*, *2* and *4* expression was measured following RNA extraction from villous tissue whereas previous studies determined SNAT1, 2 and 4 protein expression in MVM isolates from human placenta^31^.This might underlie the different findings between studies. The reduced expression of *SLC38A4* in the current study could lead to a reduction in SNAT4 expression but it is unlikely that lower SNAT4 expression exclusively accounts for reduced glutamine uptake in FGR given that it is not considered to be substrate of this isoform^42^.

The reduction in glutamine and glutamate uptake at initial rate in FGR (Fig. 1) but increase in transporter protein expression (measured in membrane-enriched placental isolates: Fig. 3) could be explained by a reduction in the insertion of the transport proteins into the MVM. Previously, reduced placental system A and L activity in FGR has been attributed to alterations in trafficking of amino acid transporters to the MVM^43^ as a consequence of a reduction in activity of the mTORC1 signaling pathway. Inhibition of mTORC1 in cytotrophoblast cells activates the ubiquitin ligase Nedd4-2^31^, which increases ubiquitination of specific isoforms of the system A and L transporters causing their removal from the plasma membrane, and a reduction in system A and L activity. This occurs without changing the overall cellular expression of these amino acid transporters. In FGR the activity of mTORC1 is reduced, and Nedd4-2 increased, compared with uncomplicated pregnancy^31^, which raises the possibility that the reduction in activity of the glutamine and glutamate transporters observed in FGR in the current study could be due to ubiquitination of the transporters and their removal from the MVM. It is possible that the raised transporter protein expression in FGR is an adaptive response in an attempt to facilitate appropriate nutrient delivery to the fetus, but these proteins fail to be inserted into the MVM. To test this hypothesis it is essential that an assessment of protein expression in MVM, rather than the mixed membrane population employed here, be conducted. The reduced glutamine and glutamate uptake following inhibition of mTORC1 by rapamycin observed in the current study shows that the activity of their respective transporters is regulated by mTORC1 in placental villous tissue of normal pregnancy. Further experiments are necessary to determine whether the reduction in glutamine and glutamate uptake in FGR is a consequence of reduced mTORC1 and increased Nedd4-2 activity lowering transporter expression in the MVM.

In conclusion, this study shows that the placental uptake of glutamine and glutamate is reduced in FGR, which may have consequences in terms of modulation of the placental amino acid pool and overall availability for transfer to the fetus. Deprivation of intracellular amino acids also compromises key aspects of syncytiotrophoblast biology such as mitochondrial function and cell renewal^44^. We also show that system X_AG-_, which transports glutamate, is regulated by mTOR in normal pregnancy.

These studies contribute to our understanding of how amino acid transport into the placenta is regulated in normal pregnancy, and also add to the wealth of evidence for compromised placental amino acid transport in FGR. Improving placental amino acid transport could be a potential therapy for FGR but further studies are required to fully understand regulatory mechanisms of the different transporters and identify appropriate therapeutic targets.

## Methods

### Tissue collection

Women who met the inclusion criteria (maternal age > 18 < 40 years; body mass index (BMI) > 19 < 30 kg/m^2^ at first antenatal appointment) were approached to take part in the study. However, difficulties obtaining tissue during the study from women with a BMI < 30 kg/m^2^ meant that some over this cut-off were included (n = 1 AGA: BMI 31.2, n = 1 FGR: BMI 33.9). Exclusion criteria were multiple pregnancy, congenital abnormalities or pre-gestational (e.g. hypertension) or gestational maternal disease (e.g. pre-eclampsia, gestational diabetes mellitus). IBR was calculated using GROW software (www.gestation.net). For the purpose of this study we defined FGR as an individualised birth weight ratio (IBR) < 5th centile, in alignment with the widely accepted threshold when this study began. AGA was classified as the 20th–80th centile to reduce the likelihood of including infants that are small or large for gestational age in the study population.

The placenta was collected immediately following delivery and trimmed placental weight (umbilical cord and placental membranes removed) recorded. Villous tissue was sampled according to a systematic sampling protocol and either used immediately for experiments requiring fresh tissue or stored at − 80 °C.

Freshly isolated villous tissue was used to compare initial rate ^14^C-glutamine and ^14^C-glutamate uptake in AGA and FGR. The benefit of this technique, compared with MVM vesicles, is the ability to assess amino acid uptake without compromising tissue integrity, intracellular signaling mechanisms and associated driving forces. The uptake of ^14^C-methylaminoisobutyric acid (MeAIB) was measured alongside as a positive experimental control. MeAIB is a non-metabolisable substrate of system A^45^ and has been used extensively to assess placental system A activity in human and animal studies^15,46,47^. Furthermore it is well established that placental system A activity is reduced in FGR versus AGA^13–15^.

### Development of a protocol to assess transporter-mediated uptake of ^14^C-glutamine and ^14^C-glutamate

A method is well established to assess system A activity in the MVM by determining Na^+^-dependent uptake of ^14^C-MeAIB into villous fragments at initial rate^45,48^. However, transporter-mediated ^14^C-glutamine and ^14^C-glutamate uptake into villous fragments has not been reported previously and a pilot experiment was performed to determine (a) the optimal concentration of radioisotope (b) a strategy to detect the transporter-mediated component of ^14^C-glutamine and ^14^C-glutamate uptake, and (c) the time over which glutamine and glutamate transporter activity could be measured at initial rate.

Glutamine transport by syncytiotrophoblast MVM vesicles is mediated by Na^+^-dependent systems A and N, and the Na^+^-independent system L^28^. ^14^C-glutamine uptake is inhibited by 5 mM histidine (substrate of system N) serine (substrate of system A) and 2-amino-2-norbornanecarboxylic acid (BCH, non-metabolisable analogue, substrate of system L)^49^. Therefore, the contribution of systems A, N and L to glutamine transport in villous fragments was measured in Tyrode’s buffer in the absence of competitive substrates (total ^14^C-glutamine uptake comprising non-specific diffusion and transporter-mediated uptake) and in control (i.e. Na^+^-containing) or Na^+^-free Tyrode’s buffer [Tyrode’s buffer as described previously^50^ with equimolar choline chloride to replace 135 mM NaCl (pH 7.4 with KOH)] with 5 mM histidine, 5 mM serine and 5 mM BCH. For ^14^C-glutamate, 5 mM aspartic acid, a substrate of the Na^+^-dependent system X_AG-_^28^ was used, as described above.

Following tissue collection (described above), villous tissue was maintained in glutamine-free DMEM (1 g/litre glucose, Life Technologies Ltd, Leicestershire, UK) supplemented with 864 μM glutamine and 120 μM glutamic acid mixed 1:1 with Tyrode’s buffer^50^, for a final concentration of 432 μM and 60 μM, respectively, to mimic the concentration in maternal plasma before beginning the experimental protocol.

To measure uptake, placental villous tissue was exposed to ^14^C-glutamine (0.066 µCi/ml; 0.24 nmol/ml) and ^14^C-glutamate (0.13 µCi/ml; 0.5 nmol/ml) for 10–120 min. After the elapsed time period, fragments were vigorously washed for 2 × 15 s in 12 ml ice-cold Tyrode's buffer (control or Na^+^-free) then suspended in 4 ml water for 18 h. Lysed tissue fragments were incubated in 0.3 M NaOH (37 °C; overnight) and protein determined on the lysate by the Bio-Rad method. Initial rate uptake was expressed per mg fragment protein, using the latter as a proxy measure of fragment size. The transporter-mediated component was determined by subtracting uptake in Na^+^-free Tyrode’s buffer containing competitive substrates from uptake under control conditions (Supplementary Fig. 1).

Analysis by linear regression demonstrated that transporter activity was at initial rate over 30–90 min. Thus, definitive experiments measured ^14^C-glutamine, ^14^C-glutamate and ^14^C-MeAIB uptake over 30–90 min (n = 14 AGA, 11 FGR) as described above and previously^45^.

To test the hypothesis that diminished amino acid uptake may lead to decreased amino acid availability for transfer to the fetus, a proxy measure of amino acid availability was calculated as amino acid uptake at 90 min (per mg placental protein) × trimmed placental weight (g).

### Effect of rapamycin on amino acid uptake at steady state

Placental villous fragments from uncomplicated pregnancies were dissected and processed under sterile conditions. Fragments were maintained in medium (DMEM 1880028: Gibco) supplemented with alanine (341 µM), glutamine (418 µM), glutamic acid (69 µM) and taurine (44 µM), streptomycin sulphate (100 mg/L), penicillin (60 mg/L) and gentamicin (1 ml/L) on Netwell permeable supports (Corning: 74 µM mesh; in a humidified incubator (37 ºC; 95 % air/5 % CO_2_)). To measure amino acid uptake at steady state^36^, fragments were incubated for 24 h with: 0.5 µCi/ml (8.5 nmol/ml) ^14^C-MeAIB; 0.066 µCi/ml (0.24 nmol/ml) ^14^C-glutamine; 0.13 µCi/ml (0.5 nmol/ml) ^14^C-glutamate; or 0.5 µCi/ml (8.5 nmol/ml) ^14^C-3-0-methylglucose (3-0MG). 3-0MG is a non-metabolised substrate of the GLUT transporters and uptake of 3-0MG was measured to assess whether rapamycin had nonspecific effects on transporters and/or an effect on placental villus tissue integrity.

After 24 h, fragments were washed (2 × 15 s with 25 ml ice cold 0.9% saline) then lysed in 6 ml 0.3 M NaOH at 37 °C overnight to release the accumulated isotope. The radioactivity of the tissue lysate and culture medium was determined as described previously^45^.

Rapamycin (Tocris, 53123-88-9) (n = 6 placentas) was added to the culture medium containing isotopes at 20–150 nM for 24 h. The effect of rapamycin on 3-0MG uptake was measured to determine whether inhibition of mTOR would affect a non-amino acid facilitated diffusion transporter. Stock rapamycin was prepared in DMSO and diluted in culture medium; equivalent DMSO (0.1%) was added to control medium.

Two approaches were adopted to determine the contribution of transporter-mediated uptake versus non-specific diffusion to the total uptake of radiolabelled amino acids at 24 h. Fragments were treated with 1 mM ouabain to block Na^+^/K^+^-ATPase activity, raise intracellular Na^+^ concentration^51^ and inhibit the activity of Na^+^-dependent amino acid transporters (systems A, N and X_AG-_). Additionally, fragments (n = 3 placentas) were incubated with a high concentration (10 mM) of unlabelled MeAIB, glutamine or glutamate to block uptake of the corresponding radiolabelled amino acids by competitive inhibition of the respective transporter proteins. Uptake in the presence of ouabain, or competing amino acids, is an estimate of non-specific diffusion.

### Placental protein expression of glutamine and glutamate transporters

Frozen placental tissue (n = 11 AGA, 10 FGR) was homogenised and centrifuged as previously described^52^. Membrane-enriched fractions were stored at − 80 °C for later Western blot analysis to detect LAT1, LAT2, SNAT5, EAAT1 and EAAT2. Proteins were separated by SDS-PAGE and transferred to Immobilon-FL PVDF membranes (Millipore UK Ltd., Watford, UK). Primary antibodies were: LAT1 (0.5 µg/ml; KE026; TransGenic Inc, Japan); LAT2 (2 µg/ml; ab75610; Abcam, Cambridge, UK); SNAT5 (1.4 µg/ml; ab72717; Abcam), EAAT1 (1 µg/ml; ab416; Abcam) and EAAT2 (2.69 µg/ml; ab178401; Abcam). β-tubulin (2 µg/ml; ab6046; Abcam) was used as a loading control. Bands detected at the predicted molecular weight (kDa) were validated by the inclusion of positive controls (MVM) during antibody optimisation. Negative controls were by omission of primary antibody. Immunoreactive species were detected with fluorescent-conjugated secondary antibodies (Li-COR Biosciences, Cambridge, UK) and membranes imaged using an Odyssey Sa Infrared Imaging System (Li-COR). Signal density was measured using Image Studio Lite (Li-COR). All signals were in the linear range of detection.

### Placental protein expression of phospho-S6K1 and S6K1 following rapamycin exposure

Placental villous explants were set up as described in the steady state measures section (n = 5). At 1, 2, 4 or 24 h, explants were taken and homogenised in RIPA lysis buffer (Sigma-Aldrich, UK, R0278) containing 10% PIC (Sigma-Aldrich, UK P8340) and 10% phosphatase inhibitor cocktails 2 and 3 (Sigma-Aldrich, UK, P5726 and P0044) using the Bullet blender tissue homogeniser (Gold units, Next Advance, USA). The mTOR signaling pathway exerts its effects via the phosphorylation of downstream targets such as ribosomal S6 kinase (S6K1). To assess mTOR activity membranes were probed, as per the Western blotting experiments above, for phospho-S6K1 (Thr-389, #9234, 84.2 ng/ml) and S6K1 (#2708, 48.0 ng/ml, both Cell Signaling Technologies, USA). All blots were re-probed with β-actin as a loading control (Sigma-Aldrich, UK, A5441, 1 µg/ml).

### Extraction of total RNA from human placental villous tissue

Total RNA was isolated from AGA (n = 11) and FGR (n = 9) villous tissue samples using the *mir*VanaTM isolation kit (AM1560, Ambion, LifeTechnologies, UK) following the manufacturer’s protocol. Samples were treated with DNAse using TURBO DNA-*free*TM kit (AM1906, Ambion, LifeTechnologies, UK) following the manufacturer’s instructions. RNA purity and concentration were assessed (Nanodrop 2000c, ThermoFisher Scientific, UK; 260/280 ratio of 2.0 ± 0.2 was considered acceptable). Samples were stored at − 80 °C. cDNA was synthesised from 500 ng RNA using an AffinityScript Multiple Temperature cDNA Synthesis Kit (Agilent Technologies, Stratagene, UK). A reference total human placental RNA (1 μg, AM7950, Ambion, Life Technologies, UK) and negative controls (no reverse transcriptase: − RT and no RNA template control: NTC) were also included. 3 μl random primers (0.1 μg/μl) was added to each tube, incubated at 65 °C for 5 min and subsequently cooled at room temperature for 10 min to allow primers to anneal to the RNA. 2 μl 10 × AffinityScript RT Buffer, 0.8 μl dNTP mix (25 mM of each dNTP), 0.5 μl RNase Block Ribonuclease Inhibitor (40 U/μl) and 1 μl AffinityScript Multiple Temperature RT were added to give a final volume of 20 μl. The reaction was incubated at 25 °C for 10 min to extend the primers, 42 °C for 60 min to generate cDNA, then terminated by incubation at 70 °C for 15 min. A pooled cDNA sample was generated by collecting 5 μl of each undiluted cDNA sample. The cDNA pool was diluted 1:4 in PCR H_2_O then serially diluted to generate samples for a standard curve (1:4–1:256). All samples were stored at − 20 °C. The efficiency of reverse transcription was checked against a housekeeping gene (TATA-box binding protein, *TBP*).

### Quantitative real-time PCR (qRT-PCR) of mRNA

A mastermix for qRT-PCR was prepared per reaction as follows: 0.2 μl PCR H_2_O, 5 μl 2X SYBR Green QPCR master mix, 0.3 μl ROX (reference dye, diluted 1:500 in PCR H_2_O) (Brilliant III Ultra-fast SYBR Green Master Mix, Agilent Technologies, Wokingham, UK) and 0.25 μl of each forward and reverse primer (final concentration 300 nM) for the gene of interest. Primer sequences were as follows: *SLC38A1* (5′-GTGTATGCTTTACCCACCATTGC-3′ and 3′-GCACGTTGTCATAGAATGTCAAGT-5′), *SLC38A2* (5′-ACGAAACAATAAACACCACCTTAA-3′ and 3′-AGATCAGAATTGGCACAGCATA-5′), *SLC38A4* (5′-TTGCCGCCCTCTTTGGTTAC-3′ and 3′-GAGGACAATGGGCACAGTTAGT-5′), and *TBP* (5′-CACGAACCACGGCACTGATT-3′ and 3′-TTTTCTTGCTGCCAGTCTGGAC-5′) used previously^45,53^. Tyrosine 3- monooxygenase/tryptophan 5-monooxygenase activation protein zeta (*YWHAZ*) primers (5′-CCTGCATGAAGTCTGTAACTGAG-3′ and 3′-TTGAGACGACCCTCCAAGATG-5′). 4 μl cDNA samples (diluted 1:10 in PCR H_2_O, 10 ng cDNA) were mixed with 6 μl master mix and measured in duplicate. A standard curve and negative controls (–RT, NTC) were included in duplicate on each plate. Conditions for qPCR (Stratagene MX3005P) were as follows: 95 °C 5 min, then 95 °C 30 s, 60 °C 30 s, 72 °C 30 s (40 cycles, amplification), 95 °C 1 min, 55 °C 30 s then a final increase to 95 °C (in 0.2 °C increments) to generate a dissociation curve; a single peak and an efficiency between 90 and 110% was considered acceptable. Cycle threshold (CT) values were interpolated from the standard curve on each plate, generated from the pooled cDNA sample to calculate mRNA levels. mRNA expression was normalised to the geometric mean of *TBP* and *YWHAZ* (stable across samples, data not shown).

### Statistical analysis

Data were analysed using GraphPad Prism 7 software; *P* < 0.05 was considered statistically significant. Normal distribution was determined using D’Agostino & Pearson omnibus normality test. Data from amino acid experiments are expressed as mean ± standard error of the mean (SEM), and least squares linear regression analyses performed to determine whether transporter-mediated amino acid uptake was linearly related to time and whether there were differences in amino acid uptake in AGA versus FGR. Western blot data were analysed using a Mann–Whitney test (transporter proteins) or 2-way repeated measures ANOVA with Sidak test (phospho-S6K1 and total S6K1). mRNA expression was analysed using a Mann–Whitney test. The effect of rapamycin on amino acid uptake was assessed by ANOVA with Dunn's multi-comparison post hoc test with values represented as median. Wilcoxon matched pairs and Mann–Whitney tests were used to assess the effect of ouabain or unlabelled amino acids on uptake, respectively.

### Ethical approval and informed consent

This study was performed with local research ethics committee approval (15/NW/0829) and in accordance with relevant guidelines and regulations. Informed written consent from all participants was obtained during pregnancy prior to collection of samples.

## Supplementary information

Supplementary Information.
