# Supplementary material for: Human placental uptake of glutamine and glutamate is reduced in fetal growth restriction
Source: Sci Rep. 2020 Oct 1;10:16197. doi: 10.1038/s41598-020-72930-7 (PMC7530652; doi:10.1038/s41598-020-72930-7)
Supplement: Supplementary file 1 — Supplementary Information. [file 41598_2020_72930_MOESM1_ESM.docx]

**Human placental uptake of glutamine and glutamate is reduced in fetal growth restriction**

Kirsty R. McIntyre*^1,2,3^, Kirsty M. M. Vincent^1,2^, Christina E. Hayward^1,2^, Xiaojia Li^1,2^, Colin P. Sibley^1,2^, Michelle Desforges^1,2^, Susan L. Greenwood^1,2^ and Mark R. Dilworth^1,2^

^1^Maternal and Fetal Health Research Centre, Division of Developmental Biology and Medicine, School of Medical Sciences, Faculty of Biology, Medicine and Health, The University of Manchester, Manchester, United Kingdom, ^2^Manchester Academic Health Science Centre, St. Mary’s Hospital, Manchester University NHS Foundation Trust, Manchester, United Kingdom, ^3^School of Medicine, Dentistry and Nursing, College of Medical, Veterinary and Life Sciences, University of Glasgow, Glasgow, United Kingdom

**Corresponding author:**

Kirsty R. McIntyre

School of Medicine, Dentistry and Nursing, University of Glasgow, Wolfson Medical School Building, University Avenue, Glasgow, G12 8QQ

[kirsty.mcintyre@glasgow.ac.uk](mailto:kirsty.mcintyre@glasgow.ac.uk)

0141 330 8025

**
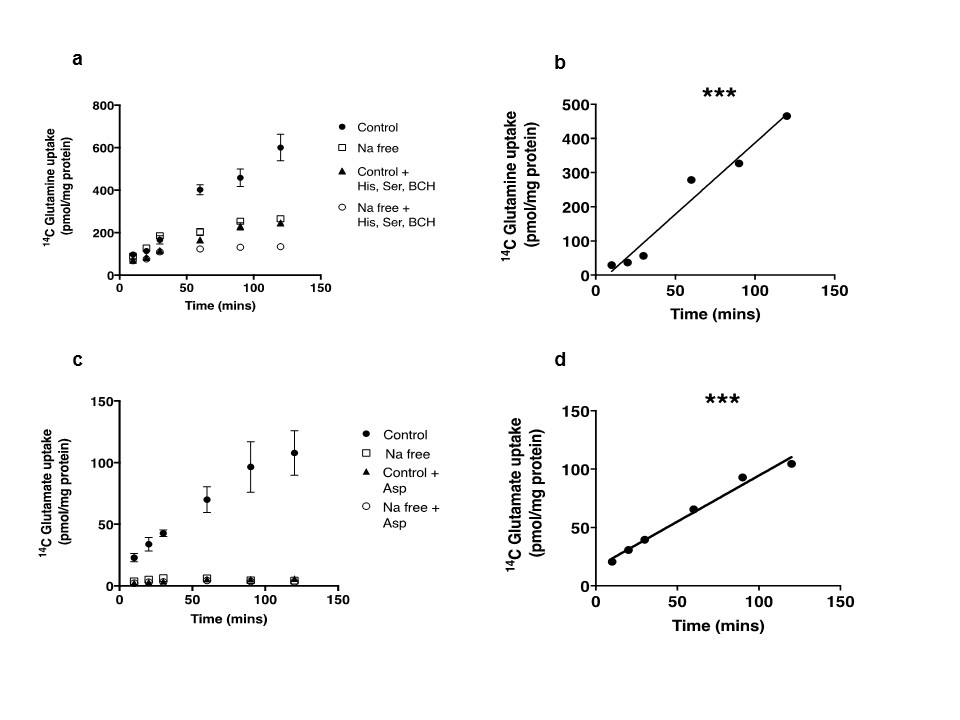

Supplementary Figure 1**
Validation of a method to determine placental transporter-mediated uptake of ^14^C-glutamine and ^14^C-glutamate into placental villous fragments. Uptake of each amino acid was measured over 10-120 min in control (Na^+^-containing) and Na^+^-free conditions ± competitive inhibitors [5 mM histidine, serine and 2-Amino-2-norbornanecarboxylic acid: ^14^C-glutamine (a); 5 mM aspartic acid: ^14^C-glutamate (c)]. Transporter-mediated uptake of ^14^C-glutamine, n = 1 (b) and ^14^C-glutamate, n = 1 (d) was taken to be the difference between uptake in Na^+^-free (non-specific diffusional uptake) and control (uptake via specific and non-specific processes) conditions. *** *P* < 0.01: Linear regression.


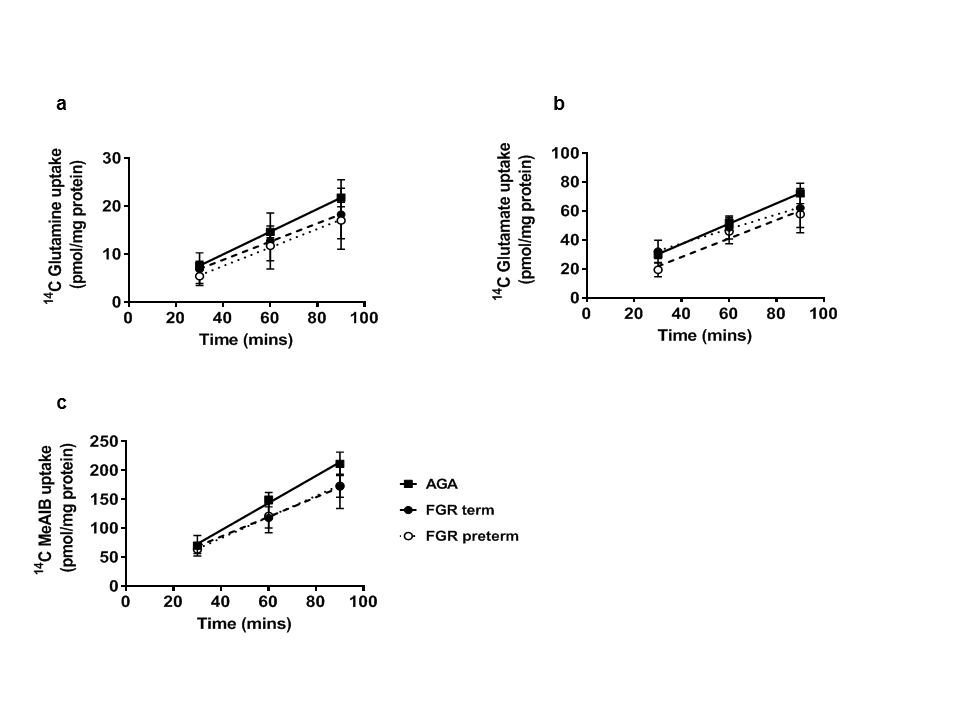
 **Supplementary Figure 2**Initial rate of ^14^C-glutamine (a), ^14^C-glutamate (b) and ^14^C-MeAIB (c) by placental villous fragments in AGA, FGR term (range: 260-284 days) and FGR preterm (204-247 days) deliveries. There was no effect of gestational age on amino acid uptake. Data are mean ± SEM; Linear regression.


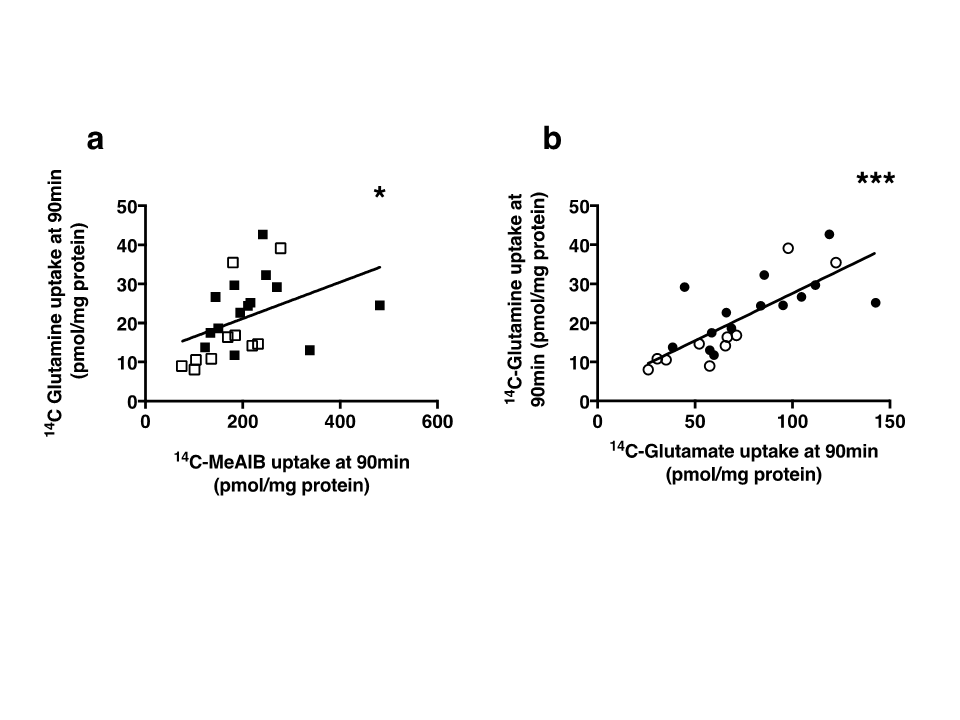
 **Supplementary Figure 3**
Correlation between ^14^C-glutamine and ^14^C-MeAIB uptake (a), and ^14^C-glutamine and ^14^C-glutamate uptake (b) by placental villous tissue in AGA (n = 14, solid symbols) and FGR (n = 10, hollow symbols) infants. * *P* < 0.05 *** *P* < 0.001 Linear regression.

 **Supplementary Figure 4**Full-length blots from Figure 3 as follows: LAT1 and β-tubulin from Figure 3A (a, b); LAT2 and β-tubulin from Figure 3B (c, d); SNAT5 and β-tubulin from Figure 3C (e, f); EAAT1 and β-tubulin from Figure 3D (g, h); EAAT2 and β-tubulin from Figure 3E (i, j).


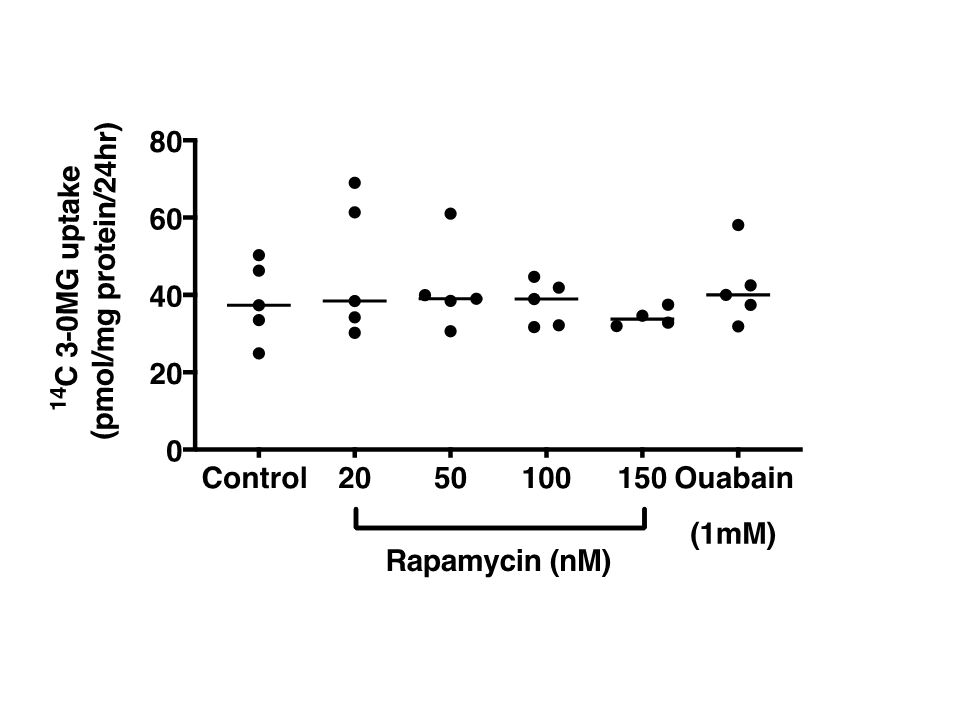
 **Supplementary Figure 5**Uptake of ^14^C-3-0-methylglucose (3-0MG) into placental villous explants (n = 5) over 24 h. Neither rapamycin (20-150 nM) nor ouabain (1 mM) had an effect on uptake (*versus* control). Line denotes median.

 **Supplementary Figure 6**Full-length blots from Figure 6 as follows: phospho-S6K1 and β-actin from Figure 6A (a); S6K1 and β-actin from Figure 6B (b).
